# Steering competitive N_2_ and CO adsorption toward efficient urea production with a confined dual site†

**DOI:** 10.1039/d3sc04688e

**Published:** 2023-10-23

**Authors:** Zhe Chen, Yonghua Liu, Tao Wang

**Affiliations:** a Department of Chemistry, Zhejiang University 38 Zheda Road Hangzhou 310027 Zhejiang Province China; b Center of Artificial Photosynthesis for Solar Fuels and Department of Chemistry, School of Science and Research Center for Industries of the Future, Westlake University 600 Dunyu Road Hangzhou 310030 Zhejiang Province China twang@westlake.edu.cn; c Institute of Natural Sciences, Westlake Institute for Advanced Study 18 Shilongshan Road Hangzhou 310024 Zhejiang Province China; d Division of Solar Energy Conversion and Catalysis at Westlake University, Zhejiang Baima Lake Laboratory Co., Ltd Hangzhou 310000 Zhejiang China

## Abstract

Electrocatalytic urea synthesis under mild conditions *via* the nitrogen (N_2_) and carbon monoxide (CO) coupling represents an ideal and green alternative to the energy-intensive traditional synthetic protocol. However, this process is challenging due to the more favorable CO adsorption than N_2_ at the catalytic site, making the formation of the key urea precursor (*NCON) extremely difficult. Herein, we theoretically construct a spatially isolated dual-site (D_S_) catalyst with the confinement effect to manipulate the competitive CO and N_2_ adsorption, which successfully guarantees the dominant horizontal N_2_ adsorption and subsequent efficient *NCON formation *via* C–N coupling and achieves efficient urea synthesis. Among all the computationally evaluated candidates, the catalyst with dual V sites anchored on 4N-doped graphene (D_S_-VN_4_) stands out and shows a moderate energy barrier for C–N coupling and a low theoretical limiting potential of −0.50 V for urea production, which simultaneously suppresses the ammonia production and hydrogen evolution. The confined dual-site introduced in this computational work has the potential to not only properly address part of the challenges toward efficient urea electrosynthesis from CO and N_2_ but also provide an elegant theoretical strategy for fine-tuning the strength of chemical bonds to achieve a rational catalyst design.

## Abstract 2

A spatially isolated dual-site catalyst manipulates the competitive N_2_ and CO adsorption to achieve efficient urea electrosynthesis, showing the theoretical feasibility of being a promising alternative to the traditional synthetic protocol.

## Graphical abstract

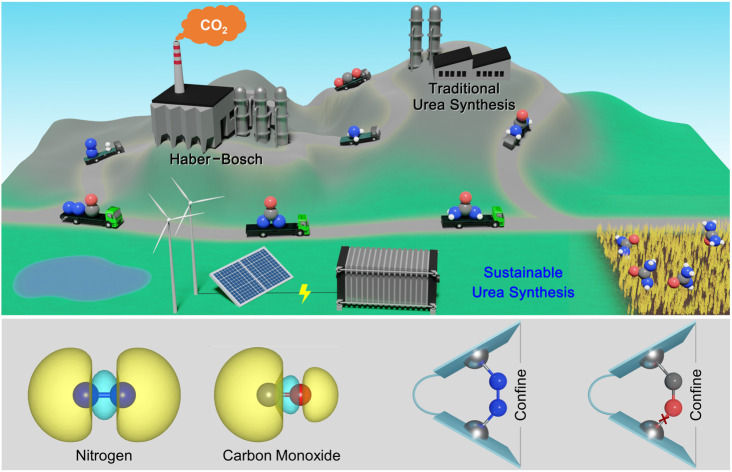

## 1. Introduction

As a crucial artificial fertilizer, urea (NH_2_CONH_2_) has laid a solid foundation for the development of agriculture to produce food and avoid the mass starvation of human beings.^1–3^ Meanwhile, it is an essential feedstock for manufacturing high-value-added chemicals such as plastics, adhesives, potassium cyanate, and urea nitrate. In industry, large-scale urea synthesis was achieved at a high temperature of 150–200 °C and high pressure of 150–250 bar using ammonia (NH_3_) and carbon dioxide (CO_2_) as reactants. Another key issue of this route lies in its dependence on NH_3_ as the nitrogen source, which is industrially produced *via* the energy- and capital-intensive Haber–Bosch process under harsh conditions.^4^ It consumes ∼2% of the global fossil energy and emits ∼300 million tons of CO_2_ annually during the inert N_2_ fixation to NH_3_ due to the usage of gray hydrogen, and ultimately impacts the sustainability of the whole urea production industry.^4^ Therefore, developing a low-carbon and alternative sustainable urea synthesis protocol is significant for achieving and maintaining the sustainable development of human society.

Recently, the direct coupling of carbon-based feedstocks (CO_2_ and CO_2_-derived CO) with NH_3_-free nitrogen sources (N_2_, NO, nitrite, and nitrate) has been proposed as a promising and efficient one-step method to produce urea *via* electrochemical reduction, which has attracted increasing attention from academia due to the sustainability and affordability.^5–20^ For example, Chen *et al.* experimentally prepared an electrocatalyst by anchoring PdCu nanoparticles on TiO_2_ nanosheets for aqueous N_2_ and CO_2_ coupling to synthesize urea, which achieved a formation rate of 3.36 mmol g^−1^ h^−1^ and a faradaic efficiency of 8.92% at −0.4 V *versus* RHE.^5^ From electrochemical CO_2_ and N_2_ coupling, Zhang and co-workers reported the urea yield rates/faradaic efficiency of 5.91 mmol g^−1^ h^−1^/12.55% and 9.70 mmol g^−1^ h^−1^/20.36% on the Mott–Schottky Bi–BiVO_4_ heterostructures and flower-like nickel borate [Ni_3_(BO_3_)_2_], respectively.^6,7^ Zhu *et al.* theoretically predicted Mo_2_B_2_, Ti_2_B_2_, and Cr_2_B_2_ as potential efficient electrocatalysts for urea production *via* N_2_ and CO_2_ coupling under ambient conditions based on the data from mechanistic calculations with the computational hydrogen electrode (CHE) model.^8^ Moreover, compared with the chemically inert N_2_ molecule, higher urea yields can be generally obtained by using more activated nitrite and nitrate, but have trouble in large-scale applications due to the limited availabilities of those nitrogen sources.^13–20^

**Fig. 1:**
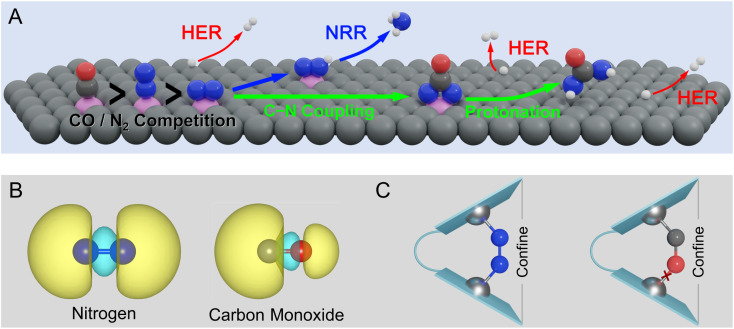
(A) Typical challenges to urea electrosynthesis by N_2_ and CO coupling from a view of the reaction mechanism. (B) HOMO electron density of N_2_ and CO. (C) Schematic illustration of N_2_/CO adsorption steering with the confined dual-site strategy.

Despite the impressive recent progress in electrochemical urea synthesis, the sluggish reaction kinetics and limited yield still seriously impede its industrial integration. In principle, the formation of key *NCON species is a prerequisite for urea synthesis,^5–7,21–23^ while the direct coupling of N_2_ with CO represents an ideal approach to generating this crucial intermediate. Unfortunately, this route faces several formidable challenges due to the intrinsic difference in electronic structures of N_2_ and CO molecules, as well as their similar bonding modes with active sites. As shown in Fig. 1A, the foremost challenge is the competitive adsorption of N_2_ and CO. Mechanistically, the binding of the polarized CO molecule with the metallic active site is usually more favorable than the non-polarized N_2_ molecule, forming a σ bond *via* the C atom due to the significant character of the highest occupied molecular orbital (HOMO) of C 2s (Fig. 1B).^24^ Meanwhile, the HOMO of the CO molecule is energetically closer to the metal d-orbital than that of the N_2_ molecule, resulting in a more stable metal-CO σ bond. As a result, N_2_ is usually a poorer π-acceptor ligand than CO,^25^ while achieving horizontal N_2_ adsorption is even more challenging. In principle, the horizontal N_2_ adsorption should be energetically ensured (Fig. 1A) to achieve the efficient formation of the *NCON intermediate *via* N_2_ coupling with CO, but unfortunately is much less competitive than CO adsorption. Clearly, this dilemma greatly restricts the feasibility of *NCON formation, leading to either CO poisoning or CO reduction to hydrocarbons during the electrochemical process. Therefore, manipulating the strong competitive CO adsorption with N_2_ at the catalytic site thereby guaranteeing the dominant N_2_ horizontal adsorption represents one of the key challenges for efficient urea electrosynthesis. Besides, as shown in Fig. 1A, even if the dominance of horizontal N_2_ adsorption is guaranteed, the competition between C–N coupling and N_2_ direct electroreduction to *N_2_H is the second grand challenge to obtain a preferential formation of the *NCON intermediate. Then, the production of urea *via* the protonation of the *NCON intermediate should be achieved at an affordable energy cost, thereby requiring high reactivity of the catalyst, which clearly denotes the third challenge. Noteworthily, the hydrogen evolution reaction (HER) is inevitable in almost all electrocatalytic processes, true also for urea electrosynthesis, which represents the fourth formidable challenge.

In this work, we proposed a strategy to properly address the aforementioned challenges by steering the competitive N_2_ and CO adsorption with a confined dual active site, which provided an elegant proposal to achieve efficient urea production from N_2_ and CO. As presented in Fig. 1C, the confined dual active site could stabilize non-polar N_2_ adsorption by forming one additional stable TM–N bond, whereas the polar CO molecule binding was hardly affected or even destabilized, thereby making horizontal N_2_ adsorption very competitive and eventually promoting the formation of the *NCON intermediate. A group of metals, mainly first-row transition metals, was chosen as active centers and anchored on nitrogen-doped graphene to construct our confined dual-site catalysts shown in Fig. 1C. Our simulations successfully predict the dual-site VN_4_ (D_S_-VN_4_) catalyst to be a very promising candidate to drive the electrochemical production of urea *via* the *NCON protonation with a low limiting potential of −0.50 V. Meanwhile, this D_S_-VN_4_ catalyst shows tremendous suppression of undesirable ammonia formation and hydrogen evolution, enabling the high reaction selectivity toward urea products. This work not only identified the spatially isolated dual site with the confinement effect for steering the competitive N_2_ and CO adsorption but also rationalized this theoretical strategy to fine-tune the strengths of chemical bonds, thus paving a promising route to obtain green urea electrosynthesis *via* rational catalyst design.

## 2. Results and discussion

### 2.1. Competitive N_2_ and CO adsorption on single and dual sites

**Fig. 2:**
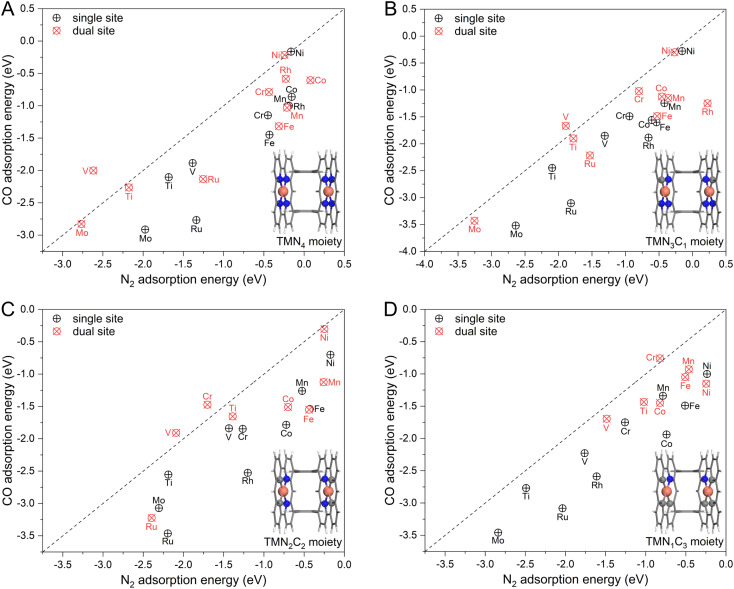
The calculated adsorption energy of N_2_ and CO molecules on the (A) TMN_4_ moiety, (B) TMN_3_C_1_ moiety, (C) TMN_2_C_2_ moiety, and (D) TMN_1_C_3_ moiety with single site and spatially isolated dual sites. The black dashed line represents the equivalent adsorption, and above the line is the N_2_-dominated adsorption region.

To achieve an efficient urea electrochemical synthesis *via* N_2_ and CO coupling, the first priority is to ensure preferential N_2_ adsorption with horizontal configuration. However, as mentioned in Fig. 1, the polar CO molecule is more likely to cover the catalytic site than the non-polar N_2_ molecule based on the analysis from molecular orbital theory. To this end, we first chose ten transition metals (TM) as active sites (Ti, V, Cr, Mn, Fe, Co, Ni, Mo, Ru, and Rh) and embedded them in graphene with different coordination environments (*i.e.*, TMN_4_, TMN_3_C_1_, TMN_2_C_2_, and TMN_1_C_3_) shown in Fig. S1.† Then, we calculated and compared their binding strengths with N_2_ and CO. As presented in Fig. 2 and the detailed energetic data in Table S1,† the N_2_ molecule energetically favors the one-sided vertical binding mode to the single active site, and the binding strength of CO is much stronger than that of the N_2_ molecule. The two exceptions, NiN_4_ and NiN_3_C_1_, with similar CO and N_2_ binding strengths are due to their physical adsorptions toward adsorbates. In general, our calculated binding strengths of CO and N_2_ on the single active site follow the vertical CO > vertical N_2_ > horizontal N_2_, which is consistent with the theoretical expectation shown in Fig. 1A. Therefore, the CO adsorption and subsequent reduction will be the dominant reaction route on the single-site catalyst if co-feeding CO with N_2_, thereby eliminating the N_2_ attacking and blocking the C–N coupling for urea synthesis.

Based on the proposed strategy in Fig. 1C, we spatially introduced a second active site with weak O binding strength but strong binding with N, namely the dual active site catalyst, to steer the competitive N_2_ and CO adsorption. Noteworthily, our recent work has successfully validated this strategy to enhance N_2_ adsorption and activation with the dual-site catalyst, making near-ambient conditions of ammonia synthesis possible.^26^ In our model catalyst shown in Fig. S2,† we sterically linked two localized single TM site catalysts by using two benzene rings. Noteworthily, this designed catalyst is very similar to the experimentally reported Pacman dinuclear porphyrins^27–30^ for O_2_ electroreduction^27^ and CO_2_ electroreduction.^28^ Thus, the adsorption of N_2_ and CO molecules was systematically examined on the designed dual-site catalysts. As presented in Fig. 2 and Table S2,† different from the cases of the single-site catalyst dominated by CO adsorption, a group of dual-site catalysts form the N_2_-dominated adsorption such as D_S_-VN_4_, D_S_-VN_3_C_1_, D_S_-VN_2_C_2_, D_S_-CrN_2_C_2_, and D_S_-CrN_1_C_3_. The *para*-configuration of the TMN_2_C_2_ moiety was also considered (Fig. S3†), which showed similar functionality and activity to the *ortho*-configuration in Fig. 2. Furthermore, we applied a Boltzmann function to evaluate the distribution of N_2_ and CO on the active sites. Our results in Fig. S4, Tables S3, and S4† clearly indicate the dominant population of N_2_ on the D_S_-VN_4_, D_S_-VN_3_C_1_, D_S_-VN_2_C_2_, D_S_-CrN_2_C_2_, and D_S_-CrN_1_C_3_ catalysts, which effectively suppresses the CO adsorption. The aforementioned results clearly reveal the capability of the designed dual-site catalyst in regulating the competitive N_2_ and CO adsorption, which lays a good basis for efficient C–N coupling.

### 2.2. Functionality of the confined dual site

**Fig. 3:**
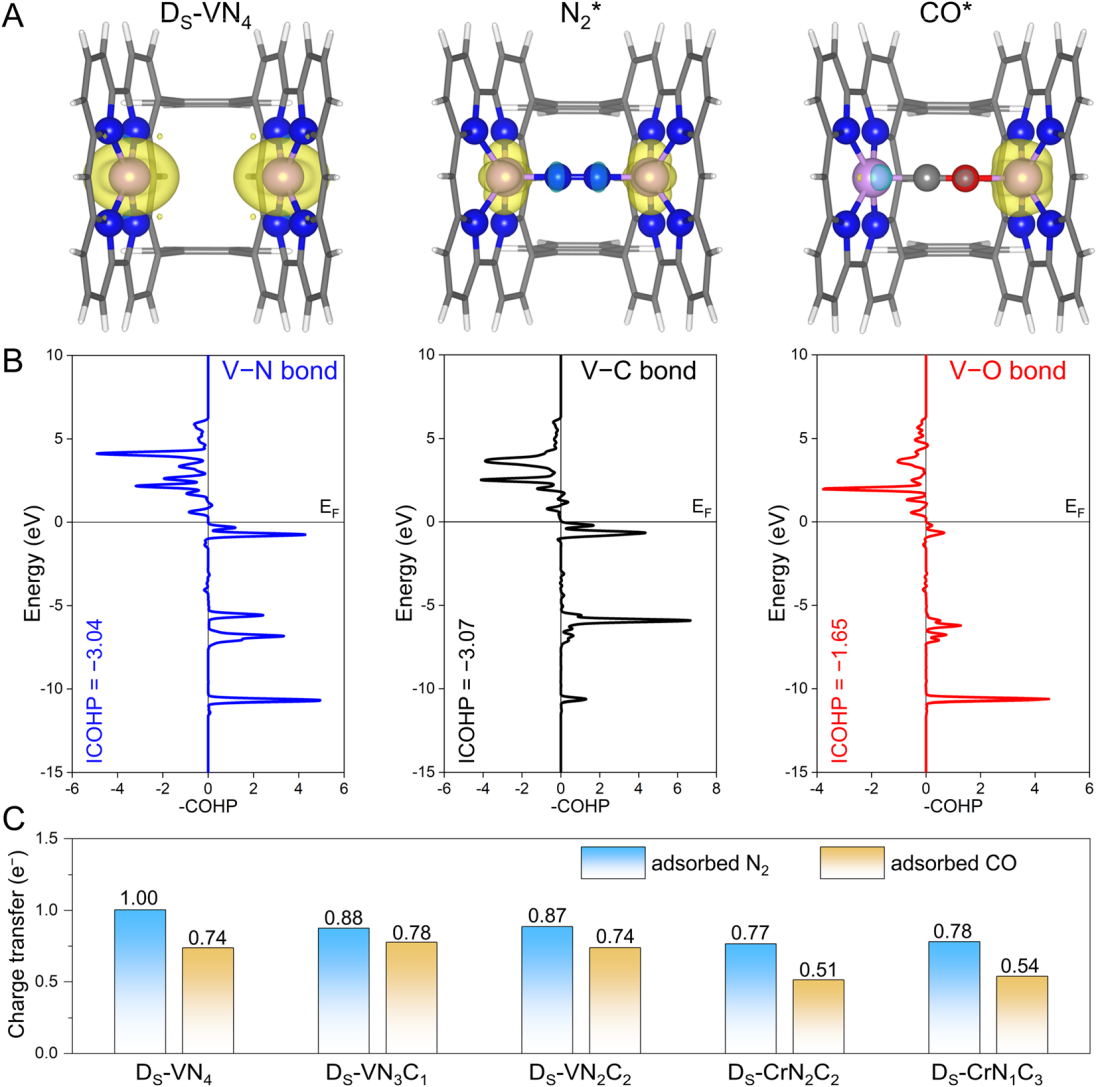
(A) The spin-charge density of pure Ds-VN_4_, N_2_ adsorption, and CO adsorption, where the isosurface value was set to be 0.009 e Å^−3^. (B) The calculated COHP between D_S_-VN_4_ and the N_2_ molecule as well as the CO molecule. The Fermi level (*E*_F_) was set to zero, and the bonding and antibonding orbitals were presented on the right and left, respectively. (C) The calculated charge transfer to adsorbed N_2_ and CO molecules.

Before moving forward to urea synthesis mechanism calculations on different confined dual-site catalysts, we further explored the origins behind these fine-tuned N_2_ and CO adsorptions. To this end, the spin-charge density maps were plotted to reveal the interactions of the adsorbates with the active sites, where the D_S_-VN_4_ catalyst was chosen as an example. As shown in Fig. 3A, the dual V site has an obviously large spin charge but gradually decreases as the adsorbates approach, indicating the involvement of spin electrons in V sites during the adsorption process. In principle, a more significant change in spin-charge represents a stronger bonding interaction. For the case of N_2_ adsorption, the V sites on both sides retain small and equal spin-charge density. For CO adsorption, the spin charge of the V site on the C atom side is negligible, while that on the O atom side is still obvious. Thus, based on the changes in the spin-charge density of the V site before and after adsorption, the bonding strength will follow the order of V–C > V–N > V–O. A similar phenomenon could be observed on the D_S_-VN_3_C_1_, D_S_-VN_2_C_2_, and D_S_-CrN_1_C_3_ catalysts as shown in Fig. S5.†

To further quantitatively evaluate the strength of chemical bonds, we performed the crystal orbital Hamilton population (COHP) analysis^31^ on the D_S_-VN_4_ catalyst. As shown in Fig. 3B, fewer bonding orbitals of V–O can be observed compared with V–N and V–C, showing a weak V–O interaction. Furthermore, the integrated COHP (ICOHP) values of V–N, V–C, and V–O bonds were calculated to be −3.04, −3.07, and −1.65, respectively. Typically, a more positive ICOHP value represents a weaker chemical bond. Thus, the strength of the chemical bonds at the D_S_-VN_4_ sites follows the order of V–C > V–N > V–O. The corresponding ICOHP values for the other four catalysts with N_2_-dominant adsorption are summarized in Table S5.† A general trend of bonding strength of TM–N > TM–O can be obtained on the designed dual-site candidates. Indeed, this trend is in line with our proposal in Fig. 1C that the sterically introduced second site can better enhance the binding strength of the N atom compared to the O atom, and eventually makes N_2_ adsorption more competitive than CO.

Meanwhile, the confined dual-site catalysts will transfer more electrons to the adsorbed N_2_ molecule *via* the additionally constructed stable TM–N bond shown in Fig. 3C. By contrast, no significant difference was found in the amount of charge transfer for the adsorbed N_2_ and CO molecules on the single-site catalysts shown in Fig. S6.† Clearly, the enhanced N_2_ adsorption and its dominance on the active site by the dual-site strategy are again supported by the electron transfer analysis. Note that the implementation of this strategy is based on the active site with strong binding to the N atom, *i.e.*, guaranteeing a strong TM–N bond. We further examined it at the single TM site by calculating the binding energy of a single N atom using the N_2_ molecule as the energy reference. As listed in Table S6,† the core active sites of the five candidates with dominant N_2_ distribution all exhibit strong N binding, implying the effectiveness of the dual-site strategy for modulating competitive adsorption. As a summary, the aforementioned spin-charge density, COHP, and charge transfer analysis provide the rationale for the fine-tuning of N_2_ and CO adsorption using our proposed confined dual-site catalysts.

### 2.3. Feasibility of confined dual-sites for urea electrosynthesis

**Fig. 4:**
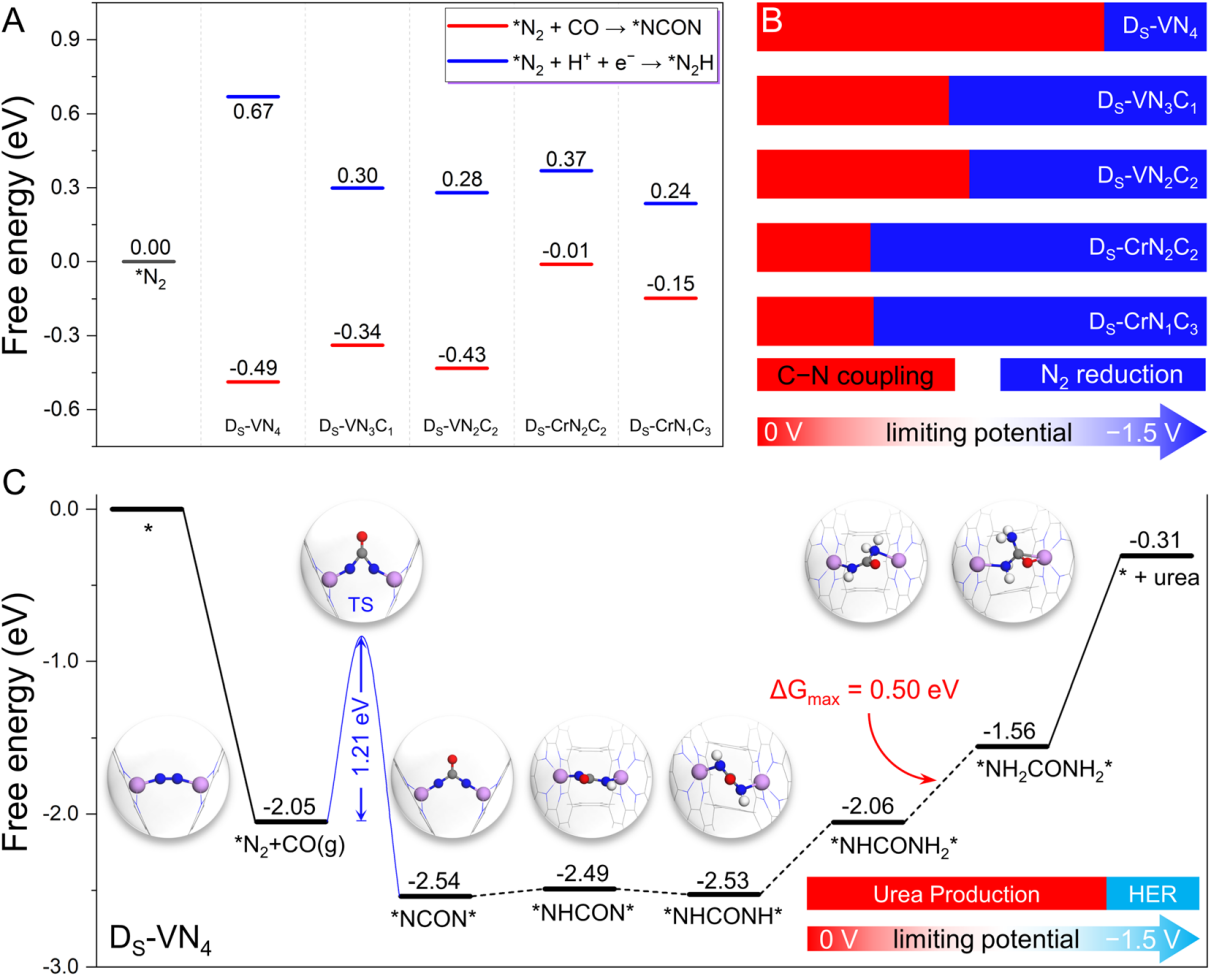
(A) The calculated free energy change of C–N coupling and N_2_ electroreduction at 0 V *vs.* RHE. (B) Reaction competition between C–N coupling and N_2_ electroreduction under the applied potential. (C) The calculated free energy diagram of urea synthesis by C–N coupling and reduction at 0 V *vs.* RHE on the designed D_S_-VN_4_ catalyst, as well as the reaction competition between urea production and H_2_ evolution under different applied potentials.

Since our proposed dual-site strategy was already able to properly address the challenge from the N_2_ and CO competitive adsorption, we further evaluated the capability of this strategy in tackling the competition between CO–N_2_ coupling and N_2_ electrochemical hydrogenation, where the five candidates with dominant N_2_ adsorption were chosen as model systems. At first, we examined the free energy changes of the coupling process *via* the Eley–Rideal mechanism (*N_2_ + CO(g) → *NCON) and the N_2_ electroreduction process (*N_2_ + H^+^ + e^−^ → *N_2_H) for convenient comparison. As shown in Fig. 4A and S7,† negative reaction free energies can be observed for the coupling process (−0.49 eV for D_S_-VN_4_, −0.34 eV for D_S_-VN_3_C_1_, −0.43 eV for D_S_-VN_2_C_2_, −0.01 eV for D_S_-CrN_2_C_2_, and −0.15 eV for D_S_-CrN_1_C_3_) on the five catalysts, indicating the thermodynamic feasibility. In contrast, the protonation of the adsorbed N_2_ molecule to the *N_2_H intermediate needs to overcome the positive free energy. Thus, at 0 V potential, the C–N coupling is thermodynamically more favorable than N_2_ electroreduction on the five dual-site catalysts.

However, the formation of the *N_2_H intermediate *via* N_2_ reduction would be electrochemically promoted by increasing the applied potential (*U*), while that of *NCON was less affected due to the noninvolvement of the proton-electron coupling step during the CO–N_2_ coupling. In other words, the free energy change of N_2_ protonation will become more negative at the high *U*, making N_2_ reduction more favorable than the C–N coupling at a certain *U*. Thus, the effect of *U* was further considered to explore this competitive process. As shown in Fig. 4A, there is a free energy gap of 1.16 eV between *N_2_H and *NCON intermediates on the D_S_-VN_4_ catalyst, which indicates that at least a *U* of −1.16 V is needed to make the formation of *N_2_H more competitive than *NCON. In principle, the D_S_-VN_4_ catalyst will preferentially form the *NCON intermediate at *U* < −1.16 V (Fig. 4B), which is named the potential tolerance limitation (PTL) in this work. Similarly, the PTL of D_S_-VN_3_C_1_, D_S_-VN_2_C_2_, D_S_-CrN_2_C_2_, and D_S_-CrN_1_C_3_ is calculated to be −0.64, −0.71, −0.38, and −0.39 eV, respectively. Therefore, the CO–N_2_ coupling process through the Eley–Rideal mechanism will be preferentially triggered unless the applied limiting potential exceeds the PTL (Fig. 4B). Besides, the possibility of adsorbed N_2_ dissociation into two isolated N atoms was also considered over these five candidates shown in Fig. S8.† Due to the significant energy requirements, N_2_ dissociation is thermodynamically very unfavorable to occur under electrochemical conditions on these model catalysts.

The above results clearly show that the five designed dual-site catalysts have the potential to achieve a high reaction selectivity for C–N coupling to form the *NCON intermediate, which could be converted into valuable urea by the subsequent electrochemical reduction process. Clearly, the D_S_-VN_4_ catalyst with the largest potential tolerance range in Fig. 4B is the most interesting candidate in our framework. Notably, the successful synthesis of a single VN_4_ site was recently reported experimentally,^32^ laying a good basis for the synthesis of the D_S_-VN_4_ catalyst.^27,28^ Therefore, we focus on the D_S_-VN_4_ catalyst to specifically explore the subsequent reaction pathway and catalytic activity for urea synthesis in the following section.

As presented in Fig. 4C, we plotted the free energy diagram of the most favorable reaction pathway for urea electrochemical production over the D_S_-VN_4_ catalyst, while the atomic structures of important intermediates were also provided. As mentioned above, the N_2_ molecule forms a horizontal adsorption mode on the dual site with two TM–N bonds, which is exothermic by 2.05 eV. Then, the formation of the *NCON intermediate from the C–N coupling occurs *via* the Eley–Rideal mechanism with a free energy change of −0.49 eV. Notably, a moderate reaction energy barrier of 1.21 eV is observed for this coupling process (Fig. S9†), which is lower than that of reported in-plane dual site catalysts (*e.g.*, 1.62 eV for Co_2_@N_6_G and 1.35 eV for FeNi@N_6_G),^21^ indicating the kinetic feasibility of generating the *NCON intermediate. Afterward, the *NCON intermediate undergoes consecutive electrochemical protonation to produce the *NHCON, *NHCONH, *NHCONH_2_, *NH_2_CONH_2_ intermediates. The corresponding free energy changes are +0.05, −0.04, +0.47, and +0.50 eV, respectively. In this case, the final protonation process (*NHCONH_2_ + H^+^ + e^−^ → *NH_2_CONH_2_) shows the largest uphill free energy of +0.50 eV, and is therefore the theoretical potential-determining step. Finally, the desorption of the *NH_2_CONH_2_ intermediate is endothermic by 1.25 eV. Theoretically, only a low limiting potential of −0.50 V is required to drive this reduction process toward urea synthesis, indicating the high activity of the designed D_S_-VN_4_ catalyst.

Due to the inevitable occurrence of the hydrogen evolution reaction (HER) in any electrochemical reactions, we further took it into consideration. Our calculated binding free energies of two H atoms on the Ds-VN_4_ catalyst (Fig. S10†) are only +0.22 eV, and much weaker than the chemisorption of the N_2_ molecule (−2.05 eV), making the binding of H atoms much less competitive than N_2_ molecules. Despite the electrochemical binding of H with the catalyst being enhanced with increasing applied potential, N_2_ adsorption will remain dominant on the D_S_-VN_4_ catalyst at *U*_L_ < −1.135 V and properly suppress the HER due to the lack of active sites to bind H. Based on the above energetic analysis of the whole reaction pathways for urea synthesis, the D_S_-VN_4_ catalyst could effectively and selectively produce urea in the limiting potential range of −0.50–−1.135 V, which are able to suppress both the N_2_ reduction and the HER. We anticipate that our proposed strategy based on confined dual-site catalysts will shed light on the experimental catalyst design with high activity and selectivity toward urea production *via* CO and N_2_ coupling.

## 3. Conclusion

In summary, we proposed a theoretically feasible strategy to steer the competitive adsorption of reactants by constructing a catalyst with a spatially isolated dual-site. With the help of the spatial formation of a more stable TM–N bond than TM–O, the interaction of the N_2_ molecule with the catalytic dual-site can be enhanced, which guarantees the effective coupling of the pre-adsorbed N_2_ with CO and facilitates the formation of the *NCON intermediate as the key precursor for urea electrosynthesis. Based on our systematic calculations on a group of transition metal-based dual-site catalysts, D_S_-VN_4_, D_S_-VN_3_C_1_, D_S_-VN_2_C_2_, D_S_-CrN_2_C_2_, and D_S_-CrN_1_C_3_ were computationally found to exhibit dominant N_2_ distribution in a horizontal mode, effectively blocking CO adsorption. Furthermore, the formation of the *NCON intermediate on these screened catalysts is thermodynamically more favorable than the *N_2_H formation from electrochemical N_2_ hydrogenation. Among all the theoretically predicted promising candidates, D_S_-VN_4_ stands out as an efficient electrocatalyst for urea synthesis with high activity and selectivity. This study demonstrates the feasibility and functionality of the confined dual-site strategy in regulating the competitive adsorption of reactants to achieve efficient C–N coupling and urea production, which can bring important theoretical guidance for the design of experimental catalysts for practical and sustainable urea synthesis.

## 4. Computational details

All the density functional theory (DFT) calculations with spin-polarization were performed using the Vienna *Ab Initio* Simulation Package (VASP) code.^33^ The revised Perdew–Burke–Ernzerhof (RPBE) functional was employed to describe the exchange–correlation interactions within the generalized gradient approximation.^34,35^ The electron–ion interactions were represented by the projector augmented wave (PAW) method.^36^ The kinetic energy cutoff of the plane wave was set to be 500 eV and the convergence criterion for the residual forces and total energies were set to be 0.03 eV Å^−1^ and 10^−5^ eV, respectively. The empirical correction in Grimme's method (DFT + D3) was adopted to describe the van der Waals interaction.^37^ The transition state with only one imaginary frequency was identified using the climbing image nudged elastic band (CI-NEB) method.^38^ Bader charge calculation was performed to analyze the charge population and charge transfer.^39^ Other computational details can be found in the ESI.†

## Data availability

Computational data supporting the findings can be found in the article and ESI,† and are available from the authors upon reasonable request.

## Author contributions

T. W. led and supervised this project; Z. C. performed all the DFT computations and data analysis; Y. H. L. contributed to the plot of Fig. 1; all authors discussed the results, and contributed to the writing of the manuscript.

## Conflicts of interest

The authors declare no competing financial interest.

## Supplementary Material

SC-014-D3SC04688E-s001
