# Supplementary material for: Steering competitive N2 and CO adsorption toward efficient urea production with a confined dual site
Source: Chem Sci. 2023 Oct 23;14(44):12707–14. doi: 10.1039/d3sc04688e (PMC10646942; doi:10.1039/d3sc04688e)
Supplement: SC-014-D3SC04688E-s001 [file SC-014-D3SC04688E-s001.pdf]

Electronic Supplementary Material (ESI) for Chemical Science.
This journal is © The Royal Society of Chemistry 2023

Steering Competitive N_2 and CO Adsorption toward Efficient Urea Production with the Confined Dual Site

Zhe Chen,[a,b] Yonghua Liu,[b] Tao Wang[b,c,d*]

[a] Department of Chemistry, Zhejiang University, 38 Zheda Road, Hangzhou 310027, Zhejiang Province, China.

[b] Center of Artificial Photosynthesis for Solar Fuels and Department of Chemistry, School of Science and Research Center for Industries of the Future, Westlake University, 600 Dunyu Road, Hangzhou 310030, Zhejiang Province, China.

[c] Institute of Natural Sciences, Westlake Institute for Advanced Study, 18 Shilongshan Road, Hangzhou 310024, Zhejiang Province, China.

[d] Division of Solar Energy Conversion and Catalysis at Westlake University, Zhejiang Baima Lake Laboratory Co., Ltd., Hangzhou 310000, Zhejiang, China.

Email: twang@westlake.edu.cn

Complement to Computational Detail

The calculated lattice constants for the single site catalyst are a = 12.78 Å, b = 12.30 Å, and the vacuum space was set to be 15 Å in the c direction to minimize the interaction between periodic images. The dual site catalyst was placed into a 20 Å cubic box for optimization. The 3×3×1 Monkhorst-Pack and the 1×1×1 Gamma-centered k-point meshes were used to sample the Brillouin zone for the single site catalyst and dual site catalyst, respectively.

The adsorption energy (E_{ads}) was calculated based on the equation: $E_{ads} = E_{total} - E_{substrate} - E_{adsorbate}$, where E_{total}, $E_{substrate}$ and $E_{adsorbate}$ represent the total energies of the systems containing the substrate and adsorbate, the substrate, and the adsorbate, respectively. According to this definition, a more negative adsorption energy indicates a stronger interaction.

The Boltzmann function was used to evaluate the distribution of N_2 and CO on the active sites, which is defined as $f_{dis} = 1/(1 + \exp\left\{-\frac{\delta G}{k_B T}\right\}) \times 100\%$, where δG is the free energy difference between N_2 and CO adsorption, k_B is Boltzmann constant, and T is the room temperature of 298.15 K.

The Gibbs reaction free energy change (ΔG) of each elementary step was calculated by using the computational hydrogen electrode (CHE) model proposed by Nørskov *et al.*[1] The chemical potential of the proton-electron pair in aqueous solution is related to that of one-half of the chemical potential of an isolated hydrogen molecule. Based on this model, the ΔG value can be obtained by the formula: $\Delta G = \Delta E + \Delta ZPE - T\Delta S + \Delta G_{pH} + eU$, where ΔE is the reaction energy of reactant and product species adsorbed on the catalyst directly obtained from DFT computations; ΔZPE and ΔS are the changes between the adsorbed species and the gas phase molecules in zero-point energies and entropy at 298.15 K, which can be calculated from the vibrational frequencies. ΔG_{pH} is the free energy correction of pH, and can be calculated by: $\Delta G_{pH} = k_B T \times pH \times \ln 10$. Notably, the pH value was set to be zero in this work for simplicity; U was the applied potential.

Table S1. The detailed calculated adsorption energies of CO and N_2 via vertical (v) and horizontal (h) mode on the designed single site catalysts. The unit is eV and the symbol # indicates unstable or physical adsorption.

Single site	Adsorption	Ti	V	Cr	Mn	Fe	Co	Ni	Mo	Ru	Rh
**TMN_4**	CO(v)	-2.10	-1.89	-1.15	-1.00	-1.45	-0.86	-0.16	-2.91	-2.77	-1.01
	N_2(v)	-1.68	-1.38	-0.45	-0.19	-0.43	-0.15	-0.16	-1.97	-1.34	-0.21
	N_2(h)	-1.68	-0.82	#	#	0.46	#	#	-1.88	-0.47	#
**TMN_3C_1**	CO(v)	-2.45	-1.85	-1.49	-1.24	-1.60	-1.56	-0.28	-3.52	-3.10	-1.89
	N_2(v)	-2.10	-1.31	-0.94	-0.42	-0.54	-0.61	-0.15	-2.64	-1.82	-0.66
	N_2(h)	-2.01	-1.29	#	-0.01	-0.04	#	#	-2.68	-1.13	#
**TMN_2C_2**	CO(v)	-2.55	-1.84	-1.85	-1.26	-1.54	-1.78	-0.70	-3.07	-3.46	-2.53
	N_2(v)	-2.19	-1.43	-1.26	-0.53	-0.43	-0.72	-0.17	-2.31	-2.20	-1.20
	N_2(h)	#	-1.21	-0.67	#	#	#	#	-2.07	-1.61	#
**TMN_1C_3**	CO(v)	-2.77	-2.23	-1.75	-1.34	-1.49	-1.94	-1.00	-3.46	-3.08	-2.59
	N_2(v)	-2.49	-1.76	-1.26	-0.79	-0.51	-0.74	-0.24	-2.84	-2.04	-1.61
	N_2(h)	#	-1.53	#	#	#	#	#	#	-1.64	-1.21

Table S2. The detailed calculated adsorption energies of CO and N_2 on the confined dual site catalysts. The unit is eV and the symbol # indicates unstable catalyst with severe deformation during the structural relaxation.

Dual site	adsorption	Ti	V	Cr	Mn	Fe	Co	Ni	Mo	Ru	Rh
**TMN_4**	CO	-2.26	-2.00	-0.79	-1.03	-1.32	-0.61	-0.22	-2.83	-2.13	-0.58
	N_2	-2.18	-2.62	-0.44	-0.21	-0.31	0.08	-0.24	-2.77	-1.25	-0.23
**TMN_3C_1**	CO	-1.90	-1.67	-1.02	-1.15	-1.49	-1.12	-0.30	-3.43	-2.22	-1.25
	N_2	-1.78	-1.89	-0.80	-0.37	-0.53	-0.46	-0.27	-3.25	-1.53	0.22
**TMN_2C_2**	CO	-1.65	-1.91	-1.47	-1.12	-1.55	-1.51	-0.30	#	-3.22	#
	N_2	-1.39	-2.09	-1.70	-0.26	-0.44	-0.70	-0.25	#	-2.40	#
**TMN_1C_3**	CO	-1.43	-1.69	-0.76	-0.93	-1.05	-1.45	-1.15	#	#	#
	N_2	-1.02	-1.49	-0.82	-0.46	-0.51	-0.82	-0.25	#	#	#

Table S3. The detailed calculated information about adsorption energy difference (ΔE), zero-point energy (ZPE), entropy change (TΔS) at 298.15 K, adsorption free energy (ΔG_{ads}), and adsorption free energy difference (ΔG) over eight dual site catalysts. The unit is eV.

**Dual site**	**TiN_4**	**VN_4**	**MoN_4**	**TiN_3C_1**	**VN_3C_1**	**VN_2C_2**	**CrN_2C_2**	**CrN_1C_3**
$\Delta E=E_{ads}(N_2)-E_{ads}(CO)$	0.09	-0.62	0.06	0.12	-0.23	-0.18	-0.23	-0.07
$ZPE(N_2)$	0.21	0.22	0.21	0.20	0.21	0.21	0.22	0.21
ZPE(CO)	0.19	0.20	0.25	0.17	0.20	0.21	0.20	0.21
$T\Delta S(N_2)$	0.10	0.08	0.09	0.10	0.08	0.08	0.08	0.09
TΔS(CO)	0.09	0.10	0.08	0.13	0.10	0.08	0.10	0.09
$\Delta G_{ads}(N_2)$	-1.63	-2.05	-2.21	-1.25	-1.34	-1.53	-1.13	-0.27
$\Delta G_{ads}(CO)$	-1.70	-1.45	-2.20	-1.41	-1.12	-1.33	-0.92	-0.18
$\Delta G=G_{ads}(N_2)-G_{ads}(CO)$	0.07	-0.60	-0.01	0.16	-0.22	-0.21	-0.20	-0.09

Note that for the values of energy difference (ΔE and ΔG), there is a small and negligible error, showing the reliable data in Figure 2 for evaluating the competitive adsorption. A more negative value of energy difference (ΔE and ΔG) indicates a stronger N_2 adsorption than CO.

Table S4. The calculated data of adsorption distributions of CO and N_2 molecules over eight dual site catalysts.

Distribution (%)	D_S-TiN_4	D_S-VN_4	D_S-MoN_4	D_S-TiN_3C_1
N_2 adsorption	5.83	100.00	61.24	0.19
CO adsorption	94.17	0.00	38.76	99.81
**Distribution (%)**	D_S-VN_3C_1	D_S-VN_2C_2	D_S-CrN_2C_2	D_S-CrN_1C_3
N_2 adsorption	99.98	99.97	99.96	97.12
CO adsorption	0.02	0.03	0.04	2.88

Table S5. The calculated ICOHP values of TM−N, TM−C and TM−O bonds over five dual site catalysts.

ICOHP	TM−N	TM−C	TM−O
D_S-VN_4	-3.04	-3.07	-1.65
D_S-VN_3C_1	-2.63	-3.07	-1.73
D_S-VN_2C_2	-2.90	-2.94	-1.68
D_S-CrN_2C_2	-2.86	-2.68	-0.94
D_S-CrN_1C_3	-2.68	-2.90	-1.31

Table S6. The calculated N affinity on the single site catalysts and the adsorption energy difference ($\Delta E = E_{ads}(N_2)$-$E_{ads}(CO)$). The unit is eV and the symbol # indicates unstable catalyst with severe deformation during the structural relaxation. A more negative value of N binding represents a stronger TM−N interaction and a more negative value of ΔE indicates a stronger N_2 adsorption than CO.

	**transition metal**	**Ti**	**V**	**Cr**	**Mn**	**Fe**	**Co**	**Ni**	**Mo**	**Ru**	**Rh**
	N binding (single site)	0.07	-0.59	-0.04	0.48	1.65	2.81	4.28	-2.49	-0.41	2.51
**TMN_4**	ΔE (single site)	0.42	0.50	0.70	0.81	1.02	0.71	0.00	0.94	1.43	0.80
	ΔE (dual site)	0.09	-0.62	0.35	0.82	1.00	0.68	-0.03	0.06	0.88	0.36
	N binding (single site)	0.22	-0.70	-0.28	0.47	0.85	2.44	3.80	-2.40	-1.42	1.84
**TMN_3C_1**	ΔE (single site)	0.35	0.54	0.55	0.83	1.06	0.95	0.12	0.88	1.29	1.23
	ΔE (dual site)	0.12	-0.23	0.22	0.78	0.96	0.67	0.03	-0.04	0.69	1.47
	N binding (single site)	0.45	-0.53	-0.52	0.37	0.51	1.91	3.64	-1.98	-1.92	0.76
**TMN_3C_1**	ΔE (single site)	0.37	0.40	0.58	0.73	1.11	1.06	0.53	0.77	1.27	1.33
	ΔE (dual site)	0.27	-0.18	-0.23	0.87	1.11	0.81	0.05	#	0.83	#
	N binding (single site)	0.86	-0.11	-0.29	-0.01	0.20	1.15	3.20	-2.30	-1.78	0.21
**TMN_3C_1**	ΔE (single site)	0.28	0.47	0.49	0.55	0.98	1.20	0.76	0.62	1.04	0.98
	ΔE (dual site)	0.41	0.21	-0.19	0.47	0.54	0.63	0.90	#	#	#

Note that there are some TM sites showing weaker N binding (*e.g.*, dual RhN_4) still present efficient improvement via dual site strategy. This may be due to the confinement introduced by the dual site catalyst that constrains the adsorption behavior of CO, making it unable to adsorb on the one-sided active site in an optimal orbital overlapping manner.

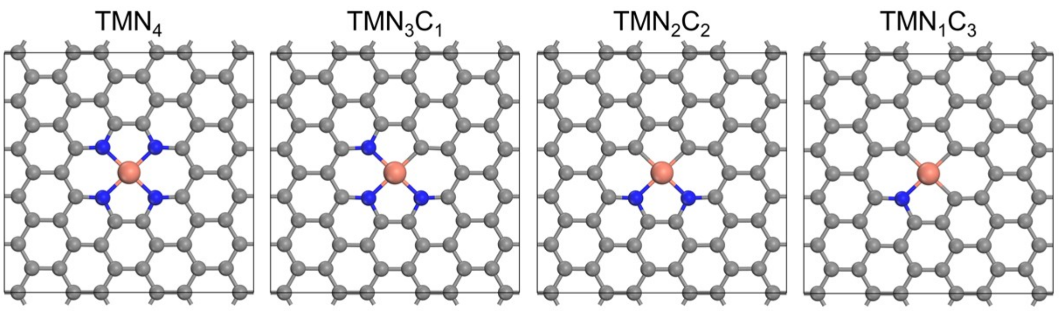

Figure S1. The detailed atomic structures of single TM atom embedded in graphene with different coordination.

Note that for the TMN_2C_2 complexes, there are two possible configurations (i.e., ortho- and para-doping). After testing, the ortho-configuration (as shown in the above Figure) is more energetically stable. Therefore, this configuration is employed herein, including single site or dual site catalysts in the main text.

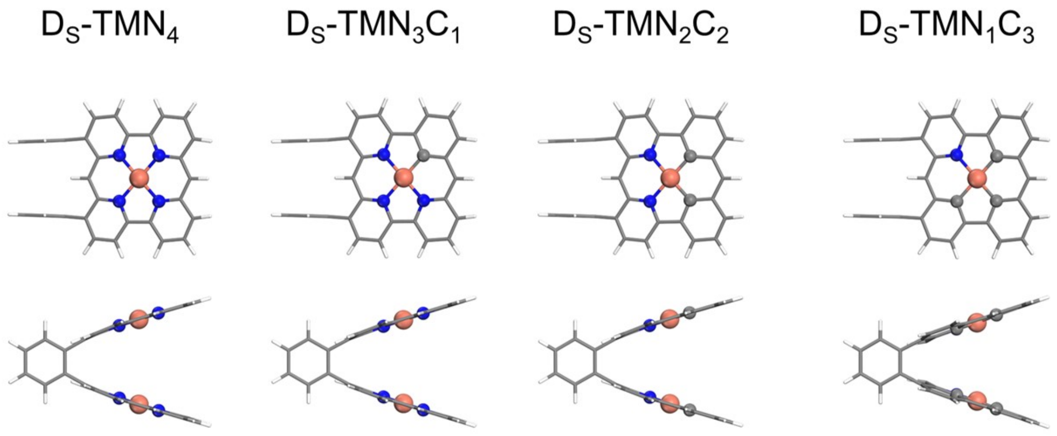

Figure S2. The detailed atomic structures of confined dual TM site.

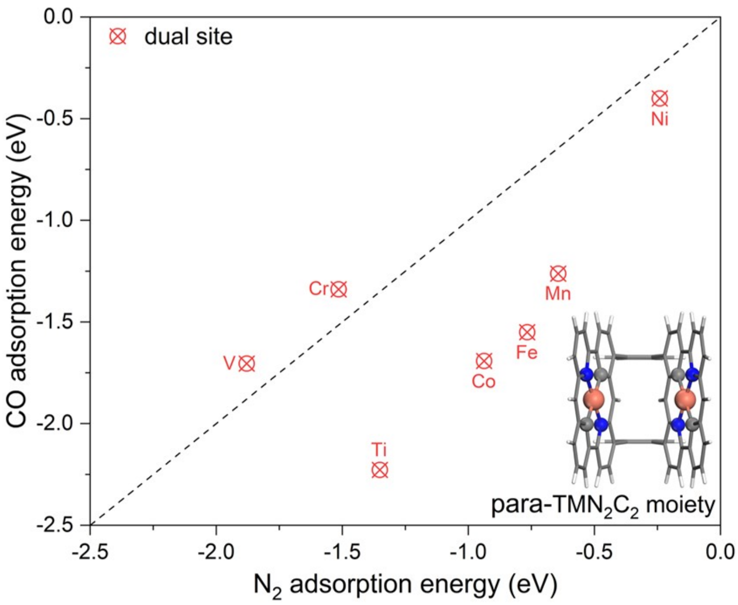

Figure S3. The calculated adsorption energy of N_2 and CO molecules on the para-TMN_2C_2 moiety with isolated single site and confined dual site. The black dashed line represents the equivalent adsorption, and above the line is the N_2-dominated adsorption region.

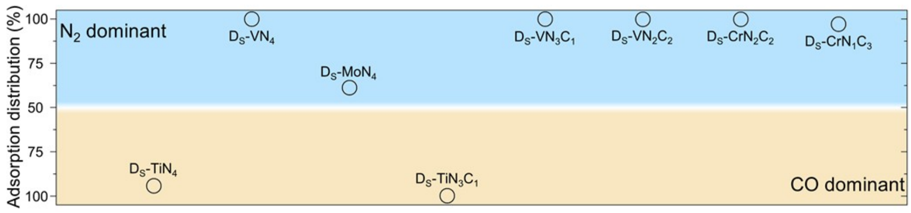

Figure S4. The calculated adsorption distribution of CO and N_2 molecules over eight dual-site catalysts.

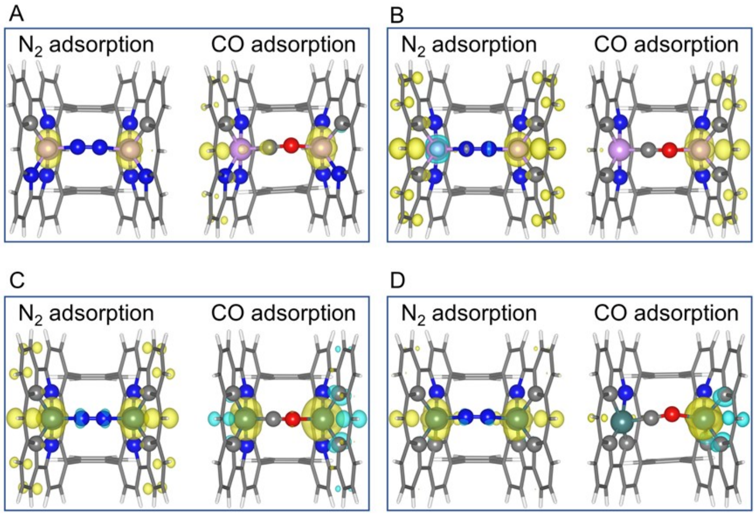

Figure S5. Spin charge density of N_2 adsorption and CO adsorption, where the isosurface value was set to be 0.009 e $Å^{-3}$ on (A) D_S-VN_3C_1, (B) D_S-VN_2C_2, (C) D_S-CrN_2C_2, and (D) D_S-CrN_1C_3.

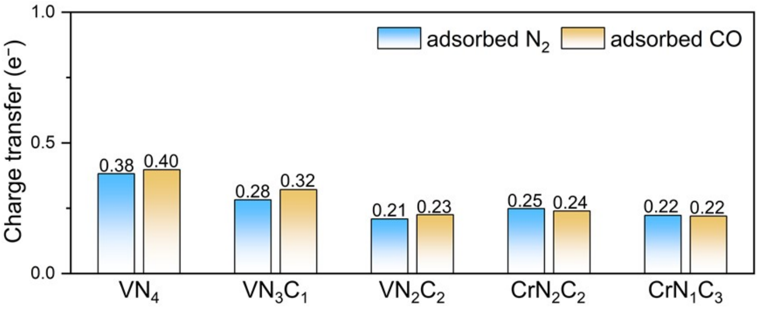

Figure S6. The calculated charge transfer on adsorbed N_2 and CO molecules on five single site catalysts.

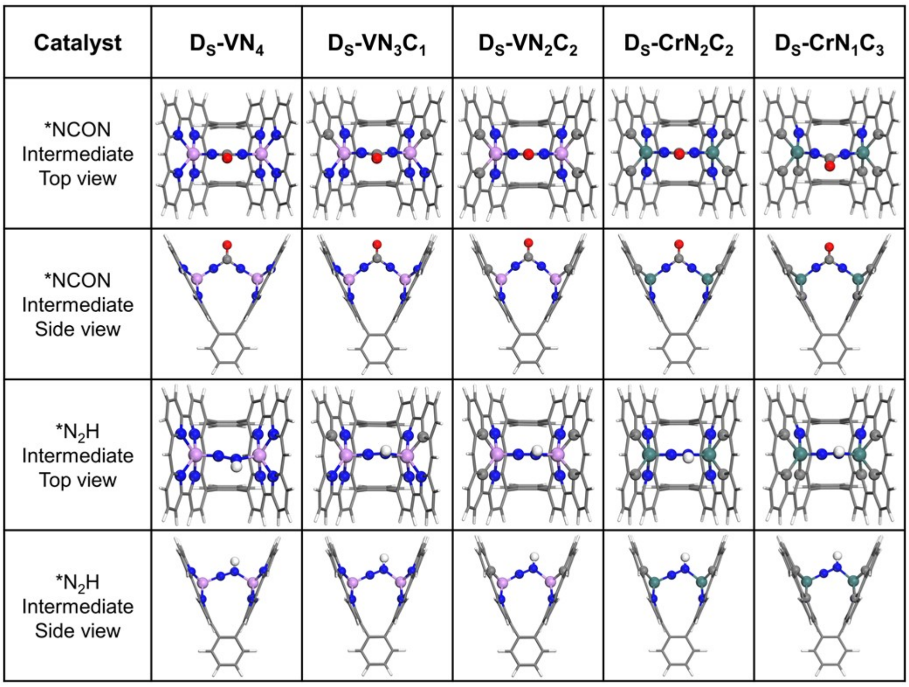

Figure S7. The atomic structures of *NCON and $^*N_2H$ intermediates over five dual site catalysts.

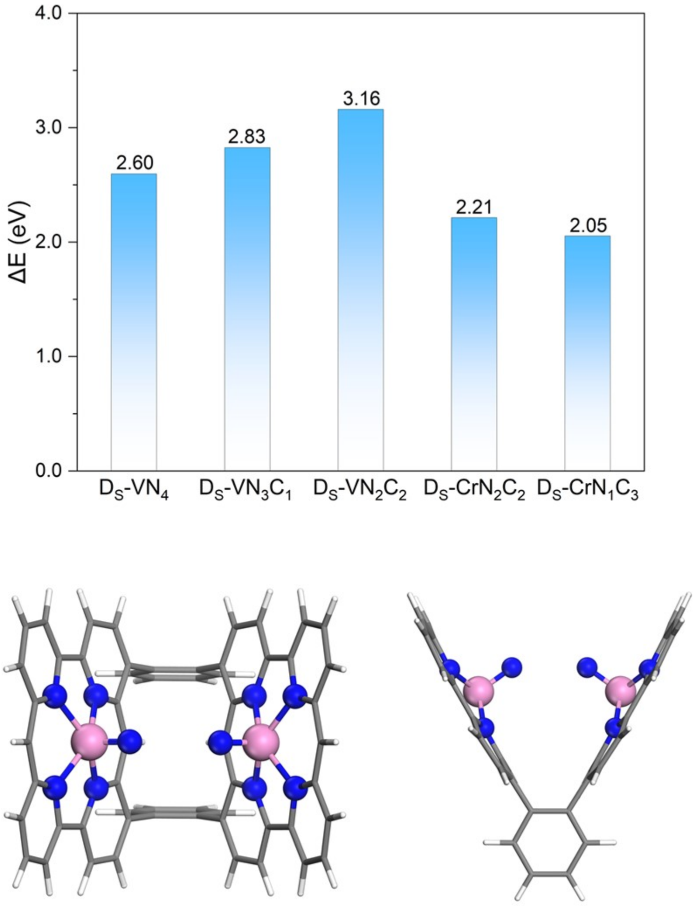

Figure S8. The calculated energy change (ΔE) from an adsorbed N_2 molecule (N_2^*) to two isolated N atoms ($2N^*$) over five candidates, and the atomic structure of two isolated N atoms adsorbed on D_S-VN_4 was given below as an example. The ΔE is defined as $\Delta E = E(2N^*) - E(N_2^*)$, where $E(2N^*)$ and $E(N_2^*)$ are the calculated total energies of two isolated N atoms and an adsorbed N_2 molecule, respectively.

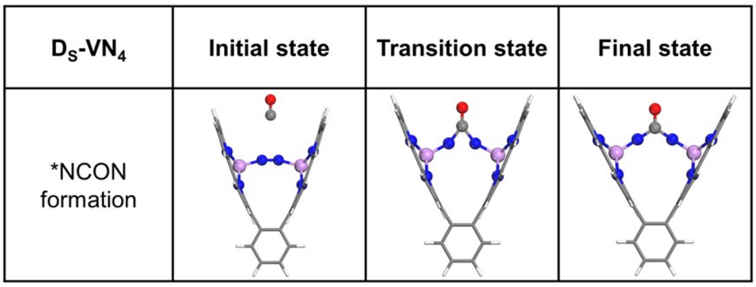

Figure S9. The atomic structures of initial, transition and final states in the kinetic pathway of C−N coupling on the designed D_S-VN_4 catalyst.

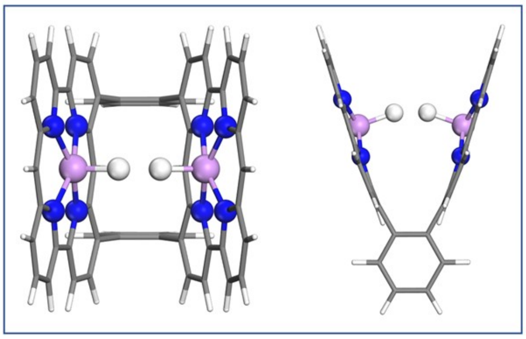

Figure S10. The atomic structures of the adsorption of two H atom on the designed D_S-VN_4 catalyst.

References

1 J. K. Nørskov, J. Rossmeisl, A. Logadottir, L. Lindqvist, J. R. Kitchin, T. Bligaard and H. Jonsson, *J. Phys. Chem. B,* 2004, **108**, 17886-17892.
